# Healthy and premature aging of monocytes and macrophages

**DOI:** 10.3389/fimmu.2025.1506165

**Published:** 2025-03-17

**Authors:** Syamantak Basu, Ying Ulbricht, Manuela Rossol

**Affiliations:** ^1^ Molecular Immunology, Faculty of Health Sciences, Brandenburg University of Technology (BTU) Cottbus-Senftenberg, Senftenberg, Germany; ^2^ Faculty of Environment and Natural Sciences, Brandenburg University of Technology (BTU) Cottbus-Senftenberg, Senftenberg, Germany

**Keywords:** monocytes, macrophages, aging, immunosenescence, rheumatoid arthritis, inflammaging, monocyte metabolism

## Abstract

Aging is associated with immunosenescence, a decline in immune functions, but also with inflammaging, a chronic, low-grade inflammation, contributing to immunosenescence. Monocytes and macrophages belong to the innate immune system and aging has a profound impact on these cells, leading to functional changes and most importantly, to the secretion of pro-inflammatory cytokines and thereby contributing to inflammaging. Rheumatoid arthritis (RA) is an autoimmune disease and age is an important risk factor for developing RA. RA is associated with the early development of age-related co-morbidities like cardiovascular manifestations and osteoporosis. The immune system of RA patients shows signs of premature aging like age-inappropriate increased production of myeloid cells, accelerated telomeric erosion, and the uncontrolled production of pro-inflammatory cytokines. In this review we discuss the influence of aging on monocytes and macrophages during healthy aging and premature aging in rheumatoid arthritis.

## Introduction

The immune system is the body’s defense against diseases and infection. As individuals age, the immune system undergoes changes that gradually impair its ability to defend against diseases, infections and other challenges; a process called immunosenescence.

Cellular senescence, stem cell exhaustion, genomic instability, telomere attrition, epigenetic alterations, loss of proteostasis, altered cellular communication, dysregulated nutrient-sensing, and mitochondrial dysfunction have been described as hallmarks of aging (1). Senescent cells secrete a variety of mediators (growth factors, proteases, chemokines, cytokines), termed the senescence-associated secretory phenotype (SASP), that affect surrounding cells and tissues (2).

In addition to immunosenescence, aging is accompanied by a chronic, low-grade inflammation. This process is called inflammaging (3). Both the innate and the adaptive immune system are altered by aging. While the innate immune system becomes more active during aging, the adaptive immune system tends to become more inactive. This leads to a decreased ability to resolve infections and establish immunological memory, as well as an increased risk of age-related diseases (3, 4).

Rheumatoid arthritis (RA) is an autoimmune disease characterized by systemic chronic inflammation, chronic infiltration of immune cells in the synovial membrane, and joint destruction. Age is considered an important risk factor for RA (5, 6) and the immune system is prematurely aged (7), making it an important model to study the molecular mechanisms of an aging immune system. RA patients have increased systemic levels of pro-inflammatory cytokines such as IL-6 and TNFα (5). Monocytes and macrophages are the main producers of TNFα and IL-6, and these cytokines are targets of the most successful therapies in RA, demonstrating that monocytes and macrophages are important players in the pathogenesis of RA. Circulating monocytes are recruited into the RA synovium via chemotaxis and this recruitment is an important factor for causing synovial inflammation (8). Monocytes also express increased cellular surface antigens, chemokine receptors, produce inflammatory cytokines and promote cartilage and bone destruction in RA (9). These immune responses contribute to RA pathogenesis. Thus, understanding the role of monocytes and how they are influenced by aging in RA can provide insight into potential therapeutic targets to modulate monocyte function or block inflammatory signals.

In this review we summarize the findings on the effects of healthy aging and premature aging in RA on the phenotype and function of monocytes and macrophages. We will focus thereby on monocytopoiesis and monocyte subpopulations, telomere length and epigenetic changes, functional consequences like cytokine production, phagocytosis, and respiratory burst, and finally on monocyte metabolism.

## Monocytopoiesis

Monocytes develop from hematopoietic stem cells (HSCs). These multipotent stem cells are located in the bone marrow and have the potential to differentiate into all blood cell types. In the myeloid lineage, HSCs then differentiate into common myeloid progenitors (CMPs), granulocyte-monocyte progenitors (GMPs) and ultimately culminate in the development of granulocytes and monocytes (10). Aging leads to changes in the differentiation of HSCs into the various blood cells (11), which results in the increased production of myeloid cells, in a process called “myeloid skewing” (12–15).

Aged healthy adults have an increased monocyte count in the peripheral blood. Reitsema et al. reported a monocyte count of 0.56 x 10^9^/L in healthy older individuals in comparison to 0.41 x 10^9^/L in young individuals (16). Similar results were reported by others (17–21). Seidler et al. observed no difference in the monocyte count (22). The discrepancies seen might depend on the different recruitment strategies of old healthy individuals and the different age spans in the cohorts. The time point of the phlebotomy might also play role in the discrepancies between the studies, especially the metabolic state and physical activity. Snodgrass et al. showed a decreased fasting monocyte count in older individuals that increased after consuming a high-fat meal (23). In addition, exercise is known to mobilize monocytes, however, the effect on the monocyte count is mostly short-term (24). In healthy mice, an increased monocyte count was observed in the bone marrow, the spleen, and the peripheral blood of old mice (15, 19, 25, 26). Aging in rats also leads to monocytosis in the peripheral blood (27).

Bone marrow progenitor cells of RA patients show an accelerated differentiation into CD14+ cells compared to control cells (28). In line with this study, Smijanovic et al. showed that the transcriptomes of bone marrow monocytes of RA patients indicate an accelerated monocytopoiesis (29). This increased production of monocytes in the bone marrow leads to an increased monocyte count in the peripheral blood and increased monocyte frequencies in the mononuclear peripheral blood cells (30–34). Klimek et al. showed that the increase in absolute monocyte numbers is more pronounced in RA patients with a high disease activity (33), and Coulthard et al. observed increased monocyte numbers in both early (<12 months disease duration) and late RA (>12 months disease duration) (34). Murine arthritis models show increased myeloid precursors in the bone marrow and peripheral blood, and increased monocyte numbers in the spleen and peripheral blood (35, 36).

In addition to bone marrow hematopoiesis, extramedullary hematopoiesis in the spleen has been reported in adults (37). Hematopoietic stem and progenitor cells (HSPCs) are able to migrate from the bone marrow via the peripheral blood into the spleen and increase myelopoiesis during infectious or inflammatory conditions (38, 39). The so-called “emergency hematopoiesis” plays a role in acute and chronic sterile inflammation (38, 40, 41). Not much is known about extramedullary hematopoiesis in the spleen during aging. Loukov et al. showed that extramedullary hematopoiesis is increased in aged mice, resulting in increased monocyte numbers (42). They also found that splenic monopoiesis is driven by TNF, suggesting that the increased extramedullary monopoiesis is caused by the chronic, low-level inflammation that occurs with age. Extramedullary hematopoiesis in the spleen has also been reported in murine arthritis models (35, 43, 44), however, studies in humans are missing.

It is known that inflammatory conditions lead to an enhanced production and release of myeloid cells from the bone marrow (45). So it’s not surprising that in both RA and aging an increased monocytopoiesis and increased numbers of monocytes in the peripheral blood are observed. TNFα and IL-1β have a profound influence on the bone marrow, already in low-grade inflammation (46, 47). Neutralization of TNFα by therapeutic antibodies led to a reduction of monocyte numbers in the peripheral blood of RA patients (48, 49), further demonstrating the role of TNFα in bone marrow mobilization.

## Monocyte subpopulations

Circulating human blood monocytes are not a homogenous cell population but can be divided into classical monocytes (CM, CD14^++^/CD16^-^), intermediate monocytes (IM, CD14^++^/CD16^+^), and non-classical monocytes (NCM, CD14^+^/CD16^++^), where CMs represent approximately 90%, IMs 5%, and NCMs 5% of peripheral blood monocytes (50). In mice, CMs are Ly-6C^++^/CD43^+^, IMs Ly-6C^++^/CD43^++^, and NCMs Ly-6C^+^/CD43^++^ (50).

Classical monocytes are released from the bone marrow and circulate in the bloodstream for approximately 24 hours (51). A similar lifespan was reported for Ly-6C^++^ monocytes in mice (52). CMs then differentiate into intermediate monocytes, however, most of the CMs leave the circulation or die. The CMs leave the circulation to enter various organs to replenish the monocyte-derived macrophage pool or migrate to sites of inflammation (53–55). Intermediate monocytes then circulate for approximately four days and all differentiate into non-classical monocytes. The NCMs circulate for approximately seven days, the Ly-6C^+^ monocytes in mice for two days (51, 52). They are known to patrol the endothelium (56, 57). All three monocyte subpopulations are able to produce pro- and anti-inflammatory cytokines. However, there are discrepancies in the literature in regard to comparisons between the subpopulations, most likely explained by different isolation strategies (57–61).

In humans, there seems to be an age-dependent dynamic in the prevalence of the three subpopulations as early as birth. However, it is important to note that monocytes in neonates originate from both fetal and adult HSCs (62), and this might influence monocyte subpopulation frequencies. Hegge et al. compared the percentage of the three subpopulations on total monocytes between healthy term neonates (mean gestational age 39 weeks) to young adults (mean age 24 years), and found a decrease of CMs and an increase of IMs in the neonates (63). Wisgrill et al. compared cord blood from premature infants (mean gestational age 29 weeks), term newborns (mean gestational age 39 weeks), and peripheral blood from healthy adults (mean age 25 years) (64). The absolute count of CMs was found to be increased in term neonates compared to preterm neonates. IMs were decreased in term neonates in comparison to preterm neonates and also in adults in comparison to both term and preterm neonates. In contrast, Sohlberg et al. and Murphy et al. observed no difference between cord blood from term newborns and blood of healthy adults, however, both did not differentiate between three subpopulations but only between CMs and IMs combined and NCMs (65, 66).

Most of the studies focus on monocytes in the peripheral blood of adults after the neonatal-to-adult hematopoiesis transition. Several studies showed that the NCM subpopulation and the IM subpopulation expand with age in adults, both in frequency and in absolute numbers (17, 67–69). In addition, several other studies reported an expansion of the NCM subset. Seidler et al. observed both an increase in NCM frequency as well as an increase in absolute numbers of NCMs in old individuals in comparison to young individuals (22). Wang et al. found an increase in NCM and a decrease in CM frequencies in old individuals compared to young individuals (70). They also observed a positive correlation of the IM frequency with age in old individuals, however, there was no significant difference between the two age groups when compared directly. Ong et al. found the absolute number of all three subpopulations increased with age (61). Mohanty et al. analyzed IMs and NCMs together, and the frequency of this combined subset was increased in older individuals, whereas CMs decreased (71).

In addition to an increase in absolute monocyte numbers, RA patients also have an expansion of the intermediate monocyte subset. Early studies reported an increase in the frequency and absolute number of CD16+ monocytes in RA patients, however, the monocytes were only classified into CMs and CD16+ monocytes (IMs and NCMs together) (72, 73). Cooper et al. also did not use the CM, IM, and NCM classification, but compared NCMs and CD14++ monocytes (CMs and IMs together) between healthy donors and RA patients (74). They observed no difference in the NCM frequency, but an increased CD16 expression in long-standing RA vs. healthy donors in the CD14++ population, most likely representing IMs. Using the CM, IM, and NCM classification, we and other groups observed an expansion of the IM subset in RA (33, 34, 58, 75–79). IMs are also the predominant monocyte subpopulation in the synovial fluid of RA patients (29, 76). While the expansion of IMs in RA is well documented, the data on NCMs are less clear. Several studies observed no difference (58, 74, 76, 79), some reported a decrease (29, 33, 80), and others an increase (34, 75). Overall, all the studies did not observe an age correlation, while in healthy adults it was shown that the NCM and IM subset expands with age, as described earlier. This suggests that there might be a premature shift in the RA patient subpopulations, at least with the expansion of the IM subset. On the other hand, most RA patient cohorts have a mean age of approximately 50 years, and although some cohorts also include younger patients, more cohorts with young RA patients are needed to analyze the influence of age on monocyte subpopulations. Disease duration seems to play no role in the expansion of monocyte subsets as the changes are already present in early RA (34, 58).

There is one other noteworthy monocyte subpopulation that is part of the classical monocyte subset, namely CD56+ monocytes (81). CD56+ monocytes were first described by Sconocchia et al. in 2005 (82). We found that the frequency of CD56+ monocytes strongly increases with age (83). Young RA patients (20-39 years old) have an increased frequency of CD56+ monocytes compared to age-matched healthy adults (83), suggesting that the monocytes of RA patients are prematurely aged. The frequency of CD56+ monocytes declined during therapeutic TNFα blockade and the decrease was also associated with a better response to the treatment. CD56+ monocytes are also expanded in obesity and SARS-CoV-2, both diseases associated with immunosenescence (81, 84, 85). Dutt et al. also showed a correlation of CD56+ monocyte frequency and age in SARS-CoV-2 patients (84).

## Telomere length

Aging is associated with telomere shortening, and Hearps et al. showed that classical monocytes and CD16+ monocytes from older individuals had shorter telomeres than monocytes from young individuals (67). Spyridopoulos et al. observed an age-dependent telomere attrition of 30 base pairs per year in monocytes (86). Hochstrasser et al. also reported a strong negative correlation between age and telomere length (87). Bone marrow-derived macrophages from old mice had shorter telomeres than from young mice (88).

Circulating monocytes do not show signs of proliferation and active cell cycle (51). This suggests that telomere shortening occurs before the monocytes enter the circulation and is already present in the myeloid precursors. Spyridopoulos et al. showed that there was a strong correlation of telomere length in monocytes, granulocytes, and CD34+ peripheral blood progenitor cells (86). Telomere length is maintained by telomerase, a reverse transcriptase able to lengthen telomeres. When telomerase components were deleted, bone marrow-derived macrophages had shorter telomeres and an inflammatory phenotype with increased oxidative stress and hyperactivation of the NLRP3 inflammasome (89).

Telomere length is associated with the risk of RA (90, 91), but most data are from T cells (92). So far, we can only speculate on telomere length in RA monocytes and macrophages. Li et al. showed that the expression of MRE11A is decreased in monocytes of RA patients (93). MRE11A is a double-strand-break repair nuclease and a decreased expression leads to telomere damage in CD4+ T cells in RA. It is also known that circulating CD34+ HSCs from RA patients have a decreased telomere length but an increased telomerase activity (92, 94). The regulation of telomerase seems to be cell-type specific in the hematopoietic system in RA and independent of disease activity, at least in naïve CD4 T cells (92). In aging, it has been shown that an increased inflammatory load is associated with increased odds for a short leukocyte telomere length (95) and inflammation can cause telomere attrition (96), but it is not clear if cytokines are the cause or the result of shortened telomere length and vice versa. It has been shown that persistent DNA damage in senescent cells initiated the secretion of inflammatory cytokines like IL-6 (97). However, more research focusing on telomere length in monocytes and macrophages in aging and RA could provide further insight into the interplay between inflammation and telomere length as the cells are one of the major producers of these cytokines.

## Epigenetic changes

Epigenetics is the structural adaptation of chromosomes that do not alter underlying DNA sequence but influence genetic function (98). Common types of epigenetic changes include DNA methylation and histone modification and can have long term effects on an individual’s health. DNA methylation patterns play a part in the determination of immune cell fate and immune responses (99). Aging leads to changes in the DNA methylation patterns, and sites with hyper-methylation and hypo-methylation have been described (100–103).

Reynolds et al. identified genes that showed an age-associated expression in monocytes, and in some of those genes the CpG methylation state mediated the association (102, 104). The described age-related methylation sites are located in enhancers linked to the expression of antigen processing and presentation genes and tend to be hypomethylated, suggesting a change in antigen presentation during aging. Saare et al. detected several CpG sites with differential methylation between age groups in monocytes (105), and they also found hypermethylation of CpG sites at the *ELOVL2* and *FHL2* gene loci. These have been previously reported to be associated with age (100). Other groups also observed age-related changes in DNA methylation in monocytes (106–109).

There have been several monocyte methylome alterations described in RA. Rodríguez-Ubreva et al. reported that the change in the methylome correlated with the disease activity score DAS28, and that the methylome of RA patients in remission resembled the methylome of healthy donors (110). They also showed that pro-inflammatory cytokines can induce the methylation pattern linked to the high DAS28 score. Mok et al. showed the hypomethylation of CYP2E1 and DUSP22 promoters in monocytes of active RA patients with erosive disease (111). Other groups also described a distinct DNA methylation pattern in monocytes of RA patients (112, 113).

The above studies show that in recent years epigenetic changes in monocytes have emerged as features of aging and RA. Currently there is insufficient data available on its functional consequences, interaction of various epigenetic modifications and their exact role in RA and aging. More insight could lead to a better predicament of therapeutic responses and opens the possibility of personalized anti-TNF treatment as demonstrated recently in a study on the PBMC methylome in RA patients using machine learning models (114). More research is also needed on the role of aging in the epigenetic modifications in monocytes observed in RA.

## Cytokines

Aging is associated with elevated concentrations of pro-inflammatory cytokines, like TNFα, IL-6, and IL-1β, in the serum (70, 115–117). There is a plethora of studies focusing on the cytokine response of monocytes and macrophages during aging and we will focus on one hand on the spontaneous cytokine secretion of resting monocytes and macrophages, and on the other hand on the cytokine response to bacterial challenges. Finally, we will discuss RA monocytes and macrophages.

### Spontaneous cytokine secretion of monocytes and macrophages

Álvarez-Rodríguez et al. reported that age is positively correlated with the intracellular expression of TNFα, IL-6, and IL-1β in circulating monocytes (117), whereas O’Mahony et al. found no difference (118). Cao et al. and Puchta et al. also did not observe a difference in the TNFα, IL-6, and IL-1β expression of monocytes in young and old individuals (17, 119). Hearps et al. however, reported an increased TNFα expression in classical, intermediate, and non-classical monocytes of older individuals (67). Pence et al. showed a decreased IL-6 gene expression in older individuals (120), corroborating the findings of Mohanty et al. who observed a decrease in IL-6+ monocytes (71). In mice, unstimulated splenic macrophages, thioglycollate-elicited peritoneal macrophages, or bone marrow-derived macrophages from young and old mice did not differ in their spontaneous TNFα, IL-6, and IL-1β secretion (121–125).

The results on pro-inflammatory cytokine secretion or expression in resting monocytes and macrophages are inconclusive. Different analysis methods (intracellular cytokine staining, ELISA of cytokines in the supernatant) and detection limits of the expected low, spontaneous monocyte cytokine secretion might play a role.

### Induced cytokine secretion of monocytes and macrophages

Cao et al. reported a higher frequency of TNFα+ monocytes, a decreased frequency of IL-6+ monocytes, and an unchanged frequency of IL-1β+ monocytes in response to stimulation with bacterial lipopolysaccharide (LPS) in older individuals (17). Puchta et al. analyzed the TNFα and IL-6 response of classical, intermediate, and non-classical monocytes of old and young individuals. They found an increased TNFα and IL-6 expression in intermediate monocytes of older individuals in response to LPS and no difference in the other two subpopulations (119). In addition, the group analyzed the LPS-induced TNFα and IL-6 secretion of isolated monocytes and observed an increased response from older individuals. An increased TNFα expression was also observed by Hearps et al. in intermediate monocytes and non-classical monocytes (67). Wang et al. analyzed the intracellular expression of IL-6 and found an increased LPS-induced IL-6 expression in all three monocyte subpopulations from aged donors (60-70 years) vs. young donors (21–30) (70). However, Pence et al. showed a decreased IL-6 gene expression in response to LPS in older individuals (120). We analyzed CD56+ monocytes, a monocyte subset that expands with age, and found that they express more TNFα in response to LPS than CD56- monocytes (83).

The findings on an increased TLR4 cytokine response is not limited to monocytes. Bouchlaka et al. differentiated peripheral blood mononuclear cells (PBMCs) into macrophages and stimulated them with LPS. They found an increased TNFα and IL-6 secretion in response to LPS in macrophages differentiated from older individuals compared to young individuals (123). Gather et al. differentiated monocytes from young and old individuals towards M1 and found no difference in TNFα and IL-6 mRNA and TNFα protein expression (126).

Whereas there is a trend towards a stronger pro-inflammatory cytokine response in TLR4 activation in monocytes and also macrophages, the response to other TLR ligands seems to be attenuated. Van Duin et al. showed that the frequency of TNFα+ and IL-6+ monocytes is diminished in older individuals when the monocytes were activated with Pam_3_CSK_4_, a TLR1/2 heterodimer ligand (127). However, they observed no difference when the monocytes were activated with lipoteichoic acid (TLR2/6 heterodimer ligand), LPS (TLR4 ligand), or flagellin (TLR5 ligand). Nyugen et al. activated whole blood with Pam_3_CSK_4_ and analyzed the frequency of TNFα+ and IL-6+ monocytes (68). IL-6+ monocytes were decreased in all three monocyte subsets, TNFα+ monocytes were decreased in intermediate and non-classical monocytes in older individuals.

LPS-stimulated thioglycollate (TG)-elicited peritoneal macrophages from old mice produced less TNFα and IL-6 than those from young mice (121). The same group also showed that activation of splenic macrophages from old mice with LPS or zymosan led to a diminished TNFα and IL-6 production (122, 128). Fallah et al. made similar observations. They reported that splenic macrophages produced less TNFα in response to LPS or killed *S. pneumoniae* (129). Gomez et al. and Chelvarajan et al. also described a decreased LPS-induced TNFα, IL-6, and IL-1β secretion in splenic macrophages from old mice (130, 131). Beharka et al. observed no difference in the LPS-induced IL-6 secretion of peritoneal macrophages between young and old mice (125). Chen and Bradley found a decreased TNFα and IL-6 secretion in resident and complete Freund’s adjuvant-elicited peritoneal macrophages from old mice, but an increased TNFα and IL-6 secretion in TG-elicited peritoneal macrophages (132). Cecílio et al. showed that resident and TG-elicited peritoneal macrophages from old mice have a decreased TNFα response (133).

The overall findings on decreased cytokine secretion are not limited to TLR4 ligands but also found with other TLR ligands. Alveolar macrophages from mature mice (10-12 months) and old mice (19-21 months) had a decreased TNFα response to ethanol-killed pneumococci and purified pneumococcal cell wall (134), and purified CD11b+ peritoneal macrophages a decreased TNFα response to zymosan (135).

The data on murine bone marrow-derived macrophages (BMDM) from old mice are less clear. Several studies reported an increased TNFα and IL-6 secretion in response to LPS (123, 133, 136, 137) Kang et al. reported an increased production of IL-1β in BMDMs from old mice in response to activation with *Staphylococcus aureus* (89). Zhao et al., however, found a decreased LPS-induced IL-6 secretion (138), whereas Mahbub et al. observed no difference in the TNFα, IL-6, and IL-1β secretion between old and young mice (128). Ramirez et al. also did not find an age effect on the LPS-induced IL-1β secretion, however, they observed a decreased response to an inflammasome activation with LPS and ATP (139). BMDMs activated with *Porphyromonas gingivalis* from older mice also showed a decreased TNFα response (140).

Published data on the anti-inflammatory cytokine IL-10 and monocytes or macrophages are scarce. Cao et al. did not observe a difference in the IL-10 expression of monocytes in young and old individuals (17). They also reported an unchanged frequency of IL-10+ monocytes in response to stimulation with LPS in older individuals. Mohanty et al. found an increased frequency of IL-10+ monocytes in older individuals (71). Boehmer et al. showed that splenic macrophages of old mice produce more IL-10 than young mice in response to LPS as well as zymosan (122), whereas Cecílio et al. observed no effect on IL-10 in LPS-activated murine BMDM (133).

While an increased TLR4-induced pro-inflammatory cytokine response was most often observed in monocytes, the cytokine response was diminished in tissue macrophages. The age effect on bone marrow-derived macrophages is also less clear than on tissue macrophages from mice. Mahbub et al. already speculated that the surrounding aging microenvironment has an effect on macrophage development (128). Gomez et al. reported similar findings (141). Inflammaging and inflammatory cytokines and chemokines secreted by senescent cells (the senescence-associated secretory phenotype, SASP) have a profound influence on surrounding cells. Gather et al. showed that age has no effect on monocyte-derived macrophages, but that an aged microenvironment in co-culture experiments with aged dermal fibroblasts drives a more pro-inflammatory macrophage phenotype (126). The aged microenvironment also drives the accumulation and activation of M1-like CD38+ macrophages in adipose tissue and liver during aging (142, 143). McQuattie-Pimentel et al. also showed that age-related changes in alveolar macrophages are defined by their microenvironment in the lung (144). Chambers et al. described the recruitment of inflammatory monocytes by senescent fibroblasts in the human skin (145).

### Cytokine secretion of monocytes and macrophages in RA

Pro-inflammatory cytokines play an important role in the pathogenesis of RA, as demonstrated by the successful therapies using TNFα and IL-6 neutralizing agents (146). We have shown that monocytes of RA patients show signs of premature aging. They express membrane TNFα and spontaneously secrete IL-1β, which leads to an increased survival of the monocytes (147). Paoletti et al. also found an increased expression of membrane TNFα on RA monocytes (148). It has also been demonstrated that the intermediate monocyte subpopulation is predominantly expanded in the synovial fluid of RA patients (29, 76) and this subpopulation is associated with aging as discussed above and characterized by a high IL-1β secretion (58, 149). Single-cell RNA sequencing of RA synovial tissue led to the identification of a *IL1B* ^+^ pro-inflammatory monocyte subset which was possibly shaped by the local microenvironment (150). Transcriptomic analysis of classical monocytes revealed that the expression of *IL6* was higher in erosive disease than in non-erosive disease in premenopausal women (median age 39 vs. 37 years) (151).

We and others have shown that monocytes of treated RA patients and healthy controls have a comparable cytokine response to LPS (147, 152, 153). However, Leirisalo-Repo et al. observed an increased TNFα secretion in LPS-activated monocytes of untreated patients with early RA (152). This is in contrast to a study by Liou (154). They reported a normal cytokine response to LPS in monocytes from untreated RA patients. We also found no difference in the IL-1β secretion in treated and untreated RA patients following monocyte activation with LPS, however, we observed a lower IL-18 response in monocytes of untreated RA patients compared to healthy controls (153). The inflammasome activation with LPS and calcium led to an increased IL-1β and IL-18 secretion in monocytes from RA patients compared to healthy controls (153).

## Phagocytosis

Aging is associated with an increased susceptibility to infections. Phagocytosis of pathogens and apoptotic cells is one of the key functions of monocytes and macrophages and plays a crucial role in immune defense.

### Human monocytes and macrophages

Published data on the effect of aging on human monocyte phagocytosis are conflicting. Hearps et al. report an impaired phagocytosis of *Escherichia coli* by human monocytes from old adults (median age 72) compared to monocytes from young adults (median age 28) (67) whereas several older publications describe no effect or a non-significant trend towards impaired phagocytosis in old individuals (155, 156). Mege et al. studied the phagocytosis of opsonized zymosan, immunoglobulin-coated sheep red cells and glutaraldehyde-treated sheep red cells (155). They observed an impaired phagocytosis of opsonized zymosan and immunoglobulin-coated sheep red cells by human monocytes from old adults (mean age 76) compared to monocytes from young adults (mean age 36) but this trend did not reach statistical significance (155). Gardner et al. found no difference in the phagocytosis and killing of *Candida albicans* in monocytes from healthy young controls (<35 years) and hospitalized patients >60 years and <35 years of age (156). Unrelated to an infectious setting, Bliederhaeuser et al. report an impaired phagocytosis of exosome-associated and free alpha-synuclein oligomers by human monocytes from old adults (median age 66) compared to monocytes from young adults (median age 24) (157). A recent study showed that monocytes and monocyte-derived macrophages from old adults (mean age 60.5) have an impaired phagocytosis of opsonized beads or *Staphylococcus aureus* in comparison to young individuals (mean age 23.7) (158).

### Murine monocytes and macrophages

Studies of aged monocytes and macrophages in mice and rats are as conflicting as in humans. The phagocytosis of latex beads by peritoneal macrophages was decreased in aged mice (85 weeks) compared to young mice (15 weeks) (159), whereas the phagocytosis of *Klebsiella pneumoniae* was increased in alveolar macrophages from old rats (18 month) compared to young rats (6 month) (160). The phagocytosis of apoptotic cells by thioglycolate-elicited peritoneal macrophages, resident peritoneal macrophages and bone marrow-derived macrophages was found to be decreased in aged mice (161–163).

Linehan et al. showed that the tissue microenvironment might play a role in age-related defects in phagocytosis (164). They reported a decreased phagocytosis of fluorescent particles by peritoneal macrophages from old mice (15-20 month) compared to young mice (8-12 weeks), whereas bone marrow-derived macrophages and bone marrow monocytes showed no age-related impairment of phagocytosis (164). Interestingly, peritoneal macrophages derived from young mice showed impaired phagocytosis when injected into the peritoneum of old mice (164). This suggests that microenvironmental factors in the aged peritoneum cause age-related defects and Linehan et al. identified IL-10 as one of the factors (164).

### Rheumatoid arthritis

Several older studies reported no difference in phagocytosis of bacteria between RA patients and healthy controls (165–167). Steven et al. observed increased phagocytosis of opsonized *Staphylococcus aureus* and *Proteus mirabilis* by monocytes from RA patients compared to age-matched controls (168). The phagocytosis rate correlated positively with age when patients and controls were analyzed combined, and the phagocytosis rate of RA patients remained higher than in controls in the 30-60 age group (168). More recent studies also did not focus on age. Fragoulis et al. observed no difference in the phagocytosis of secondary necrotic cell remnants in RA patients and controls (169) and Tas et al. found no difference in the phagocytosis of apoptotic cells in monocyte-derived macrophages from RA patients and controls (170).

Interestingly, Lee et al. reported that activated platelet-induced CD16 (also known as FcγRIII) on classical monocytes participates in increased antibody-dependent cellular phagocytosis (171). The monocytes then resemble intermediate monocytes, a monocyte subpopulation expanded in the blood and synovial fluid of RA patients (58, 76) and reported to be expanded in older individuals (17, 23).

## Respiratory burst and reactive oxygen species

As described earlier, immune functions such as phagocytosis decline with age. Phagocytosis is accompanied by a sudden increase in the activation of oxidative metabolism known as the respiratory burst, which is responsible for killing phagocytosed pathogens, and mediated by the NADPH-oxidase (172). The enzyme catalyzes the formation of superoxide by transferring one electron to oxygen from NADPH (173). Other prominent reactive oxygen species (ROS) are hydrogen peroxide and hydroxyl radical. ROS are not only produced in response to pathogens or pathogen-associated molecular patterns by the NADPH-oxidase, ROS are also formed during mitochondrial oxidative phosphorylation and by the 5-lipoxygenase (173).

### ROS and respiratory burst

Aging leads to a decrease in ROS produced during the respiratory burst in monocytes and macrophages. Alvarez et al. have shown that the production of ROS decreases with age in rat peritoneal macrophages (174, 175). Phorbol Myristate Acetate (PMA)-activated 12-month-old rat macrophages showed a lower production of superoxide ions compared to 3-month-old rats. Macrophages from 24-month-old rats had a further reduction in ROS production compared to both 12-month-old and 3-month-old rats. Activation of macrophages with N-formylmethionyl-leucylphenylalanine (fMLP) or concanavalin A did not show a decrease in ROS production between 3 and 12-month-old rats, however, a significant reduction in 24-month-old rats compared to both 3 and 12-month-old rats was observed (175).

The PMA-induced superoxide generation in human monocytes is negatively correlated with (176) and the PMA-induced capacity to produce superoxide ions is reduced in aged subjects (65-75 years old) compared to young (25-35 years old) and mature (45-55 years old) (177). Lower ROS production was also reported in alveolar macrophages. Ganguly et al. measured the production of superoxide in alveolar macrophages from female Fisher-344 rats stimulated by opsonised zymosan and found that aged rats showed lower ROS production (178). One limitation of this study was the use of aged breeder rats which have been shown to exhibit a lower magnitude of respiratory burst compared to young and aged virgins (179). Esposito et al. compared alveolar macrophages obtained from virgin adult and aged female rats and observed that normal senescent mice released larger quantities of superoxide when activated by either PMA or zymosan (180), contrary to previous findings.

### ROS accumulation in aging monocytes and macrophages

In contrast to the ROS produced in activated monocytes and macrophages, the general ROS content in resting monocytes and macrophages seems to increase with age. Sebastián et al. showed an increased ROS content in BMDM from old mice compared to young mice (88). Jacinto et al. reported an increased ROS content in circulating monocytes from aged mice (181). Their study focused primarily on the apoE-/- mouse model of spontaneous atherosclerosis, but they also observed a significant increase in ROS content in monocytes from 18-month-old mice compared to 2-month-old wild-type mice.

Saare et al. reported similar findings in human monocytes (105). Oxidative stress due to ROS production was determined by measuring the fluorescence of oxidized products of chloromethyl derivative of 2’,7’-dichlorodihydrofluorescein diacetate (CM-H_2_DCFDA) in monocytes isolated from young (mean age 29.6 years) and old (mean age 79.4 years) donors (105). Unstimulated monocytes from older donors showed a twofold increase in ROS production, while LPS-stimulated monocytes showed a smaller but still significant increase. Higher cellular levels of ROS are associated with DNA damage (182), and Saare et al. observed that double-strand breaks were 1.5 times higher in the old cohort by analysing the percentage of monocytes showing a γH2AX signal (105).

Wang et al. also reported an increased ROS content (measured with DCFH-DA) in all three monocyte subpopulations (CM, IM, NCM) from elderly individuals (60-70 years) compared with those from young persons (21-30 years) (70). Hayashi et al. observed a correlation of monocyte ROS content (measured with dichlorofluorescein and hydroethidine) and age in combined exposed and unexposed Hiroshima atomic bomb survivors (65-95 years) (183). The monocyte ROS content also correlated with the radiation dose implying that atomic bomb radiation accelerates immunological aging. In our study on CD56+ monocytes, a monocyte subpopulation associated with aging, we found increased ROS content (measured with dihydrorhodamine 123) in CD56+ monocytes compared to CD56- monocytes (83).

### ROS and rheumatoid arthritis

Monocytes in RA are under high oxidative stress compared to healthy controls as shown by Ostrakhovitch and Afanas’ev (184). Oxygen radical production of RA monocytes and healthy controls were measured by chemiluminescence with luminol or lucigenin. PMA-stimulated RA monocytes showed 2.7 times more oxygen radical production compared to controls. Monocytes from RA patients also showed an increase in NADPH oxidase activity compared to healthy controls. In addition, oxygen radical production was decreased in monocytes from RA patients in the presence of mitochondrial inhibitors rotenone and antimycin A, while normal cells were unaffected, suggesting that mitochondrial superoxide production is another source of oxygen radicals in RA monocytes.

Other studies also show that superoxide anion production is increased in monocytes from RA patients compared to healthy controls. Repo et al. showed that monocytes from untreated, early RA patients showed significantly higher Luminol-enhanced chemiluminescence to stimulation by FMLP, PMA or opsonised zymosan particles compared to healthy controls (185). Hurst et al. observed an increased superoxide anion production in RA monocytes stimulated with IgG-treated zymosan or serum-treated zymosan (186, 187). No difference in basal rates of monocyte superoxide anion production was observed between controls and RA patients. Mateen et al. showed that RA patients have an increased ROS formation compared to age-matched controls. They stained ROS with 2’, 7’-dichlorofluorescein-diacetate (DCFH-DA) to observe the intracellular ROS production (188). Nitric oxide (NO), an important component of inflammatory oxidative burst, was also shown to be 1.56 times higher in monocytes isolated from the blood of RA patients compared to healthy individuals (189).

## Monocyte metabolism

The bioenergetic status of monocytes and macrophages determines cellular functions, with pro-inflammatory macrophages relying on glycolysis and the pentose phosphate pathway and anti-inflammatory macrophages relying on oxidative phosphorylation and fatty acid oxidation (FAO) (190). Monocytes and macrophages can undergo metabolic reprogramming when challenged with a stimulus and switch from an aerobic profile to anaerobic glycolysis in a process called the Warburg effect. Aging impairs oxidative phosphorylation and FAO pathways and this shift might contribute to inflammaging (191, 192).

### Glycolysis and oxidative phosphorylation

Maoldomhnaigh et al. compared macrophages differentiated from monocytes from adult blood (aged 18-69 years) and cord blood (term neonates) (193). Both MDMs responded with a rapid increase in glycolysis when activated with LPS, but only the MDMs from adults showed a decrease in oxidative phosphorylation.

Wang et al. reported an increased glycolysis, impaired oxidative phosphorylation, and mitochondrial dysfunction in all three monocyte subsets in aged adults (70). These changes were most prominent in non-classical monocytes, and the authors speculated that the increased glycolysis is a result of the mitochondrial dysfunction to meet the demand for energy metabolism.

Reduced cellular respiratory capacity with age has been attributed to dysfunctional mitochondria (194). Saare et al. also reported lower mitochondrial membrane potential despite higher mitochondrial mass in aged subjects (105). The oxygen consumption rate (OCR), basal and maximal of monocytes from young (mean age 29.6 years) and old (mean age 82.3 years) individuals were measured to calculate the spare respiratory capacity (SRC) which was significantly lower in the older group. Pence and Yarbro also reported impaired mitochondrial respiratory activity in classical monocytes from old individuals using flow cytometry (120). Their monocyte isolation strategy included CD16 depletion to only focus on classical monocytes. Both studies showed that basal respiration remains largely unaffected by age but the maximal respiratory capacity is reduced in monocytes of older individuals which leads to lower SRC.

Additionally, gene expression studies in monocytes of young (average age 35.7) and old individuals (average age 71.6 years) also highlighted the downregulation of oxidative phosphorylation genes *PLA2G4B* and *ALOX15B* in older individuals, while *PDK4* which inhibits pyruvate dehydrogenase complex was highly upregulated. This correlates to a shift of glucose metabolism from oxidative phosphorylation to lactate production in monocytes of older individuals (195). Interestingly, the NADPH oxidase, the enzyme involved in respiratory burst, is not affected by age (177).

However, Pence and Yarbro did not find conclusive evidence of increased glycolysis in LPS-stimulated aged monocytes ex-vivo (196). The microenvironment plays a significant role in age-related changes which could be a reason for such an observation. As the authors point out, there could be several epigenetic modifications and alterations in sirtuin activity that could be linked to metabolism.

### FAO and lipid metabolism

There is an increase in circulating fatty acids in older adults since aging leads to the redistribution of body fat from subcutaneous to visceral, the latter being less efficient in the storage of fatty acids (197). Additionally, Wang et al. reported decreased FAO and increased presence of lipid droplets (LD) in aged monocytes of mice and humans (198). They also observed that PPAR-α expression was downregulated in monocytes of aged humans (>60 years old) and subsequently were able to prove that this caused the FAO decrease and LD increase by downregulating PPAR-α in young monocytes to similar effects. This also affects monocyte polarisation as the accumulation of LD in aged monocytes leads to a pro-inflammatory phenotype.

### NAD+ metabolism

As an important regulator of metabolism, NAD+ is thought to also undergo changes during aging. Endogenous NAD+ is depleted during aging which eventually disrupts cellular metabolism (199). NAD+ can be obtained by several pathways, the two most commonly studied ones being *de novo* biosynthesis pathway, also called kynurenine pathway (KP), or the salvage pathway through nicotinamide precursors. Clement et al. showed that the NAD+ precursors remain stable with age, while NAD+ levels are reduced (200). This suggests that the decline in NAD+ might be caused by an increase in the activity of NAD-consuming enzymes. It is currently not clear which pathway is primarily responsible for providing NAD+ in macrophages as previous reports show conflicting results based on the characteristics of the macrophage studied. Liu et al. showed that *de novo* synthesis majorly occurs in the liver (201). However, Minhas et al. showed that decreased cellular NAD+ can also be replenished by macrophages via *de novo* synthesis (202). Disruption of the salvage pathway using a NAMPT inhibitor in human monocyte-derived macrophages (MDMs) demonstrated a marked increase in KP enzymes and metabolites. They also confirmed that basal *de novo* NAD+ synthesis accounts for 40% of total NAD+, and almost all when the salvage pathway is blocked by the NAMPT inhibitor FK866. Furthermore, they also showed that loss of the KP disrupts mitochondrial respiration by inhibiting KP enzymes *IDO1* and *QPRT*. Peritoneal macrophages from *IDO1-* or *QPRT-*deficient mice showed reduced cellular NAD+ amounts compared to wild type controls, along with suppressed oxygen consumption and increased glycolysis. This effect was reversed on supplementation with kynurenine. Similarly, human MDMs also showed a decreased NAD+ and mitochondrial respiration when IDO1 and QPRT were inhibited. Additionally, loss of QPRT activity also elevated lactate, pentose phosphate pathway intermediates and pro-inflammatory TCA intermediates that favour IL-1β and IL-6 production. Analysis of QPRT expression in human MDMs derived from young (<35 years old) and older (>65 years old) individuals showed a significant decline in older macrophages, the older macrophages having a metabolite profile similar to that observed in LPS-stimulated macrophages. Suppression of NAD+ synthesis in aged mice indicated that the KP derived NAD+ is critical in sustaining the cellular concentration. The age-associated loss of this pathway likely contributes to the proinflammatory shift of macrophages with age.

Macrophages after inflammation, however, seem to be dependent on the salvage pathway for NAD+. Cameron et al. observed that murine BMDM showed reduced NAD+ levels when stimulated by LPS compared to resting M0 macrophages or IL-4-stimulated macrophages (203). NAMPT inhibition by FK866 treatment induced dose-dependent cell death in LPS-stimulated macrophages while the viability of M0 and IL-4 macrophages was affected to a lesser extent. Inflammatory cytokines such as TNFα, IL-1β, IFNγ induce the rate limiting enzyme for the salvage pathway, NAMPT. The authors reported that this is necessary to counter the rapid NAD+ depletion on LPS activation. As mentioned earlier, these cytokines also increase with age which likely enables the macrophages to keep up with the demand due to an increase in NAD+ consuming enzyme activities resulting from glyceraldehyde-3-phosphate dehydrogenase activity and Warburg metabolism.

### Metabolic reprogramming in rheumatoid arthritis

Several studies on RA monocytes and macrophages show a pro-inflammatory metabolic profile. Metabolomic profiling showed an increased presence of lactic acid, citrate and succinate in the synovial fluid of RA (204). Shime et al. showed that lactic acid promotes pro-inflammatory pathway (205). Succinate too is known to be induced on LPS activation, as a result of the metabolic shift from oxidative phosphorylation to glycolysis during inflammation leading to its accumulation as a TCA cycle intermediate. These suggest that the macrophages in the synovial fluid might show a pro-inflammatory phenotype. Michopoulos et al. showed that itaconic acid is a marker of RA using the Tg197 mouse model which correlates with findings from Lampropoulou et al. who reported that itaconate is involved in the regulation of succinate levels in LPS-stimulated mouse bone marrow-derived macrophages (206, 207).

Yoon et al. described that Solute carrier family 7 member 5 (SLC7A5) is increased in RA patients (208). LPS-stimulated human peripheral monocytes from RA patients showed a 5.85-fold increase in SLC7A5 expression compared to healthy controls. LPS-mediated expression was also observed in human macrophages. They further reported the role of SLC7A5 in being involved in the glycolytic shift and production of pro-inflammatory cytokines such as IL-1β. McGarry et al. (30) showed that RA monocytes have increased mitochondrial respiration and enhanced glycolysis after activation with LPS. Blockade of glucose consumption resulted in an inhibition of the release of pro-inflammatory mediators. They also reported that RA monocytes have an increased number of mitochondria and an increased mitochondrial respiration already *ex vivo* without any stimulation. These above studies show that monocytes in RA tend to have a pro-inflammatory metabolic profile, and thus RA patients exhibit signs of premature aging.

## Conclusion

**Figure 1:**
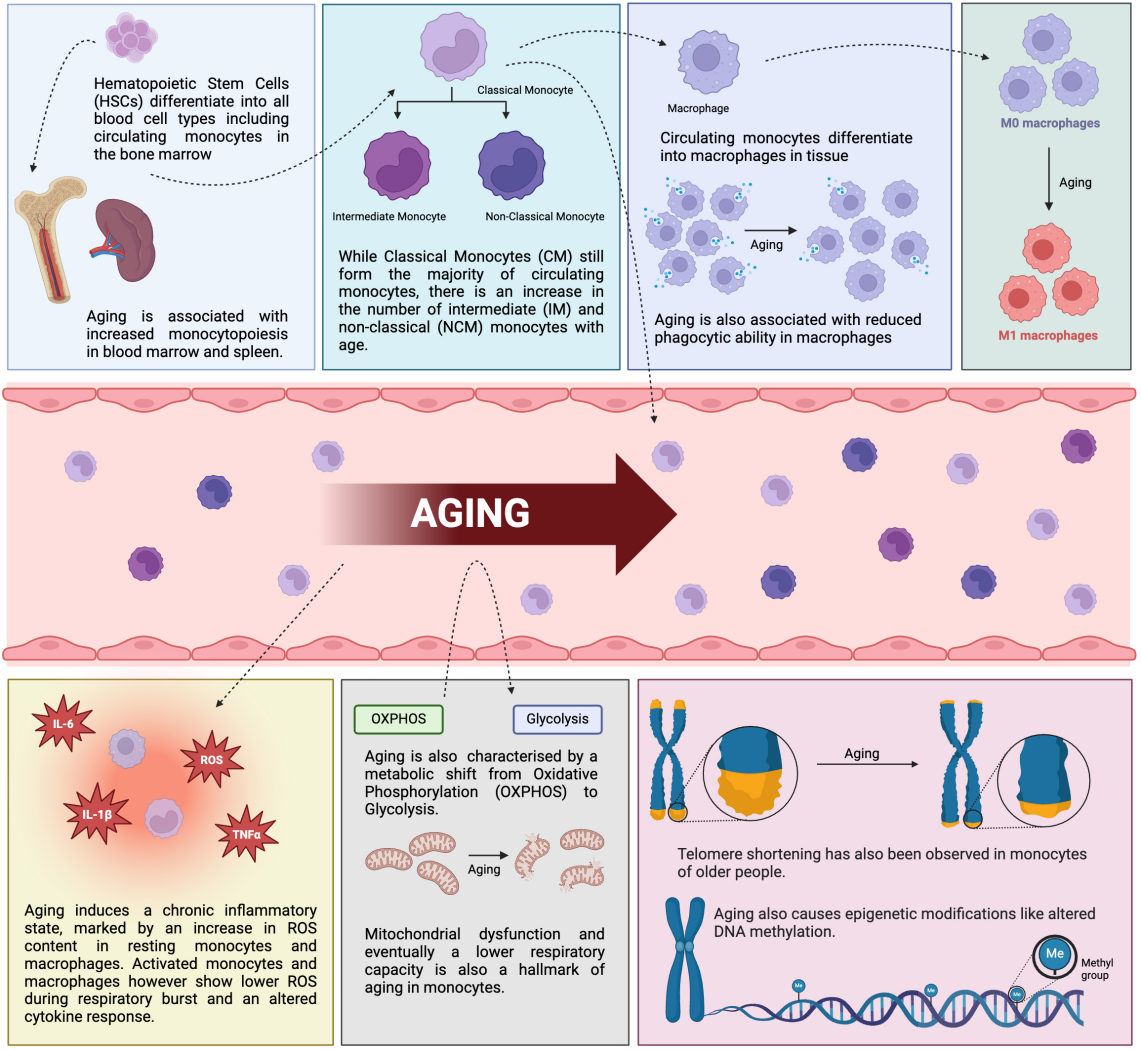
Impact of healthy aging on monocytes and macrophages. Schematic diagram displaying the consequence of aging on the phenotype and functions of monocytes and macrophages in healthy individuals. IL-6, interleukin-6, TNFα, tumor necrosis factor α, IL-1β, interleukin-1β, ROS, reactive oxygen species (created in BioRender.com).

**Figure 2:**
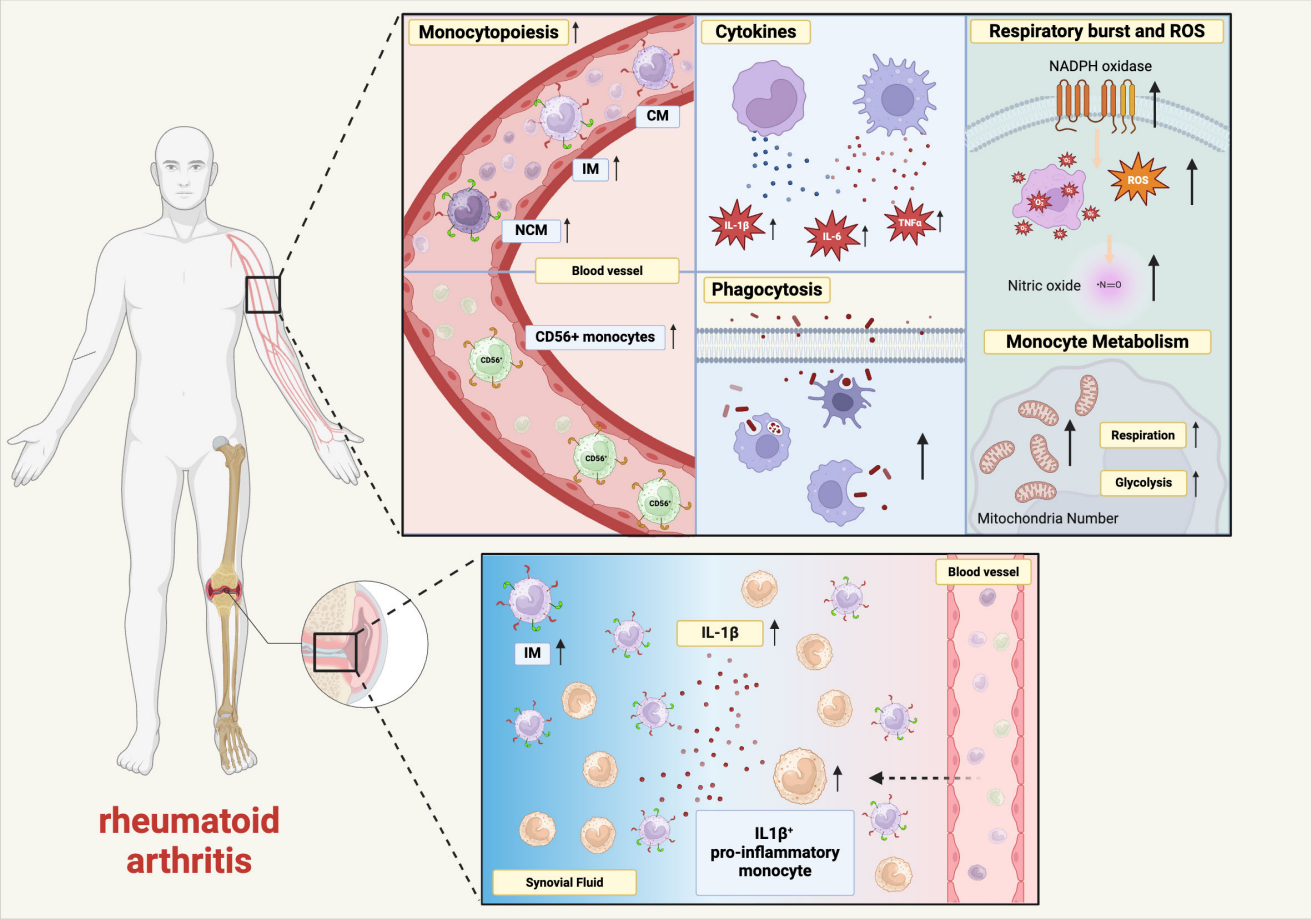
Premature aging in monocytes and macrophages in rheumatoid arthritis. Schematic diagram showing the effect of RA on age-related changes in monocyte and macrophage phenotypes and functions. IL-6, interleukin-6, TNFα, tumor necrosis factor α, IL-1β, interleukin-1β, ROS, reactive oxygen species, CM, classical monocytes, IM, intermediate monocytes, NCM, non-classical monocytes, NADPH, nicotinamide adenine dinucleotide phosphate (created in BioRender.com).

Monocytes and macrophages as part of the innate immune system are markedly influenced by aging. The myeloid biased hematopoiesis leads to increased numbers of monocytes in the peripheral blood, and the monocyte subpopulations are shifted towards intermediate and non-classical monocytes during healthy aging as well as in rheumatoid arthritis. Monocyte and macrophage functions like phagocytosis and cytokine production are affected by aging. The findings on healthy aging of monocytes and macrophages are summarized in **Figure 1** and aging of monocytes and macrophages in RA in **Figure 2**. However, further research is needed as there is still inconclusive data on the functional consequences of healthy aging and premature aging in RA on monocytes and macrophages.
